# Participant experiences of mindfulness-based childbirth education: a qualitative study

**DOI:** 10.1186/1471-2393-12-126

**Published:** 2012-11-13

**Authors:** Colleen Fisher, Yvonne Hauck, Sara Bayes, Jean Byrne

**Affiliations:** 1 School of Population Health, The University of Western Australia, Perth, Australia; 2 Curtin University and King Edward Memorial Hospital, Curtin Health Innovation Research Institute, Perth, Australia; 3 Research Implementation Fellow, Collaboration for Leadership in Applied Health Research and Care - Nottinghamshire, Derbyshire and Lincolnshire, University of Nottingham, England, UK; 4 Adjunct Midwifery Research Fellow, Curtin University, Curtin Health Innovation Research Institute, Perth, Australia; 5 Honorary Research Fellow, Curtin University, Curtin Health Innovation Research Institute, Perth, Australia

**Keywords:** Childbirth, Antenatal education, Mindfulness, Childbirth satisfaction, Qualitative

## Abstract

**Background:**

Childbirth is an important transitional life event, but one in which many women are dissatisfied stemming in part from a sense that labour is something that happens to them rather than with them. Promoting maternal satisfaction with childbirth means equipping women with communication and decision making skills that will enhance their ability to feel involved in their labour. Additionally, traditional antenatal education does not necessarily prepare expectant mothers and their birth support partner adequately for birth. Mindfulness-based interventions appear to hold promise in addressing these issues. Mindfulness-based Child Birth Education (MBCE) was a pilot intervention combining skills-based antenatal education and Mindfulness Based Stress Reduction. Participant experiences of MBCE, both of expectant mothers and their birth support partners are the focus of this article.

**Methods:**

A generic qualitative approach was utilised for this study. Pregnant women between 18 and 28 weeks gestation, over 18 years of age, nulliparous with singleton pregnancies and not taking medication for a diagnosed mental illness or taking illicit drugs were eligible to undertake the MBCE program which was run in a metropolitan city in Australia. Focus groups with 12 mothers and seven birth support partners were undertaken approximately four months after the completion of MBCE. Audio recordings of the groups were transcribed verbatim and analysed thematically using the method of constant comparison by all four authors independently and consensus on analysis and interpretation arrived at through team meetings.

**Results:**

A sense of both ‘empowerment’ and ‘community’ were the essences of the experiences of MBCE both for mothers and their birth support partner and permeated the themes of ‘awakening my existing potential’ and ‘being in a community of like-minded parents’. Participants suggested that mindfulness techniques learned during MBCE facilitated their sense of control during birth, and the content and pedagogical approach of MBCE enabled them to be involved in decision making during the birth. The pedagogical approach also fostered a sense of community among participants which extended into the postnatal period.

**Conclusions:**

MBCE has the potential to empower women to become active participants in the birthing process, thus addressing common concerns regarding lack of control and satisfaction with labour and facilitate peer support into the postnatal period. Further education of health professionals may be needed to ensure that they respond positively to those women and birth support partners who remain active in decision making during birth.

## Background

Childbirth is an important transitional life event, yet there is a large body of literature reporting maternal dissatisfaction with labour and childbirth experiences [1-10]. In part, this dissatisfaction stems from a sense that labour is something that happens to women, rather than with them [7,9,10]. Preliminary research also suggests that maternal dissatisfaction with the experience of childbirth and intrapartum care can be associated with symptoms of acute or post-traumatic stress [11]. Additionally, studies suggest that much of what women hear from family and friends hinders, rather than improves their experiences of childbirth and early parenting [12].

Promoting maternal satisfaction with childbirth, then, is linked to women accessing relevant information to make informed choices rather than simply consenting to decisions relevant to their labour and birth [13-15]. Being able to make informed choices means more than women gaining access to factual information. Research suggests that women should be equipped with communication and decision making skills that work to enhance their ability to feel part of the experience of labour [12]. Similarly, it has been recognised that existing childbirth education classes do not adequately equip women with skills and information necessary for early parenting [16].

In addition to neglecting skills acquisition, traditional childbirth education does not actively promote prenatal mental health, both of expectant mothers and their birth support partners. This is of concern due to the relationship between prenatal mental health and postnatal maternal outcomes and foetal development; as well as child development [17-19]. Given these findings, it is apparent that childbirth education should emphasise both a skills-based approach [12,20] and promote mental health for both expectant mothers and their partners.

One approach that holds significant promise in promoting psychological resiliency is the use of mindfulness-based interventions [21,22]. Mindfulness involves the cultivation of moment-to-moment awareness of experience with a non-judgmental attitude. Interventions using mindfulness have been shown to be beneficial in preventing psychological dysfunction across a range of people [21] including prenatal women and their partners. Thus, combining mindfulness with a skills-based approach to childbirth education holds promise for enhanced outcomes for expectant mothers and their partners [22].

Mindfulness Based Childbirth Education (MBCE) is a novel combination of skills-based antenatal education and Mindfulness Based Stress Reduction (MBSR). It is an eight week, one session per week program and was run as a pilot intervention by a childbirth educator who is also a specialist antenatal yoga and mindfulness meditation teacher. Each session lasted approximately 2.5 hours and was for both expectant mothers and their birth support partner. Participants had homework CDs related to mindfulness meditation and were encouraged to undertake daily practice of techniques learnt. Readings related to antenatal education were also assigned, including review articles from the Cochrane Library. Results of its feasibility and outcomes of the MBCE protocol for first time mothers are reported elsewhere (Unpublished manuscript, Byrne, Hauck, Fisher, Bayes & Schutze). Participants’ experience with the classes is the focus of this paper.

## Methods

A generic qualitative approach [23] using focus groups was utilised for this component of the study.

### Participants

To be included in the study, pregnant women (between 18 and 28 weeks gestation) had to be over 18 years of age, healthy, nulliparous with singleton low-risk pregnancies and not taking medication for a diagnosed mental illness or taking illicit drugs. The study took place in a metropolitan city in Australia with participants recruited through a range of strategies including newspaper articles, online pregnancy forums, email lists and flyers at hospitals, birth centres and organisations that offer antenatal education classes. Eighteen expectant mothers (aged 21 – 37 years (mean 30.06, SD= 3.69)) and their birth support partners completed MBCE. Sixteen expectant mothers were married or living with a partner, 14 were tertiary educated - including four with a postgraduate qualification, eight planned to use obstetrician-based care, three opted for midwife-based case and seven chose some form of shared care. Ten delivered in a private hospital. In terms of characteristics of labour, 10 were spontaneous with 11 births by caesarean section. All women reported establishing breastfeeding after birth. Twelve new mothers and seven birth support partners (6 partners and one new mother’s mother) participated in the focus groups which occurred approximately four months after completion of the program to ensure all participants were at least six weeks post-birth.

### Data collection and analysis

Two focus groups lasting approximately forty five minutes were undertaken – one for mothers and one for their birth support partners. It was important to obtain the perspective of both the mother and the birth support partner as their experiences and perceptions of the program may have differed. Concurrent but separate groups ensured that both members of each mother/birth support partner dyad felt confident to speak openly, particularly if their perceptions and experiences differed from each other. The focus groups were facilitated by experienced focus group facilitators (YH and CF). Written consent to participate and for the audio recording of the group was obtained from each participant.

Following verbatim transcription, data were analysed thematically using the method of constant comparison [24-26]. As such transcripts were read line by line, and units of meaning identified and coded. As data analysis proceeded, these units of meaning were coded onto major categories of meaning and abstracted to thematic level. To ensure rigour in analysis, all four authors analysed the transcripts separately and met as a team to ensure consensus in analysis and interpretation.

Ethical approval to undertake the study was received from the Human Research Ethics Committee of the University of Western Australia.

## Results and discussion

A sense of both ‘empowerment’ and ‘community’ were the essences of the experiences of MCBE for mothers and their birth support partners. The interactive and experiential delivery of the MBCE program facilitated the empowerment of each individual and couple during the birthing process. Additionally, among the group a sense of community was fostered and maintained, and extended beyond the MCBE sessions and birth of participants’ babies. Notions of ‘empowerment’ and ‘community’ thus permeate the themes identified during data analysis for this study which were labelled ‘awakening of my existing potential’ and ‘being in a community of like-minded parents’. Although, for the purpose of clarity, the themes are presented discretely, they are inextricably linked in dynamic and complex ways. Direct quotes have been used throughout to illustrate how participants’ experiences support the themes, and numbers (i.e. mother 1 or birth support partner 3) have been used to ensure confidentiality.

### Awakening of My existing potential

‘Awakening of my existing potential’ relates to the feelings and expectations, both of the mothers and their birth support partners, about their competence, abilities and understandings of what would be required of them during childbirth. These feelings and expectations also pattern their reflections on the MBCE sessions and of labour and birth. Participants reflected on their feelings prior to attending MBCE and described how they wanted to fulfil their respective roles during the birthing process, but did not have a real understanding of exactly what that role might entail. As one mother stated:

…at that point of my pregnancy which was two months … , I don’t feel I had the knowledge and … I had no expectations of what the course was actually going to be about and so it was just improving, building my knowledge and giving me the strength and ability to be in control of the situation and so it [attending MBCE] was. just more about building that understanding of what it all meant to be pregnant and to have a baby and all those sorts of things. (mother 3).

Prior to commencement of MBCE, birth support partners in particular, described being unaware of exactly what their role during the birthing process would be. The feelings of birth support partners are exemplified in the following quote:

I came in [to begin the MBCE program] thinking “what do I need to know?” But yeah I didn’t have… expectations of … all this stuff I can learn and I was sort of just blissfully unaware of my role. (birth support partner 3).

Our finding is consistent with that of Johnson [27] who found little support for men having clear ideas about the possible roles they will undertake during birth. The role of men has been previously described in the literature as a ‘teammate’ or ‘supporter’ for their partners – a role which facilitates that of the midwife but renders them passive - responding to their partners’ request for support and offering moral encouragement [28] and that of ‘coach’ [29,30]. Johnson also notes that, although support people initially have confidence that they can meet the demands of their role, they quite often find it more arduous than they had anticipated [27]. The birth support partner’s description above of being ‘blissfully unaware’ of what his role may entail, foreshadows that he may have underestimated the intensity of the role he was to perform during the birth of his child.

For an experienced birth support partner, (the mother of a pregnant woman), awakening of existing potential was not related to being unaware of what her role in the birthing process may entail, rather it was a reminder, or in her words, a ‘rekindling’ or a remembering of knowledge of options and experiences during birth:

I’ve always been aware of…alternatives [for childbirth] and things like that, but I think it was just…rekindling those sorts of things, those feelings I’d had. (birth support partner 6).

An important part of awakening existing potential for the mothers was the development of confidence within themselves and their body’s capacity to birth:

I think my expectations changed throughout the course as well… My expectations upon myself to have a labour and deliver this baby, you know, feeling very empowered. So it was really great to see… as we went along and just the confidence I think has grown in the group and by the end of it [MBCE] everyone was like “yes, you can do this”. So it was very transitional I think from the outset. (mother 2).

During their actual labour and birth, the mothers’ confidence was realised through their using the techniques learned during MBCE to stay in the moment and maintain a sense of control. One mother describes it as follows:

I wouldn’t have thought I could have done this [birthed], but after [MBCE] ] I knew I could do it and I was doing this [mindfulness] and I just found it very good to be with myself at this stage [during labour]. (mother 4).

Despite both mothers and their birth support partners benefiting from MBCE, however, it was the women who were the drivers behind their attendance.

For me it went “oh yeah” sure, I’ll do this [attend MBCE sessions] ‘cause I kind of have to. (birth support partner 4).

The use of the term ‘drag’ was quite commonly used, both by mothers and birth support partners, as the means by which the mothers ensured attendance by their birth support partners, reflecting common perceptions of how the decision was made to attend:

I actually had to drag my husband along to the first session here, but after the first session he was wanting to come. So that was good (mother 7).

After initial attendance, birth support partners as well as the mothers could see the real value in attending MBCE:

 I just found it [MBCE] incredibly useful and valuable and I’m so glad I got dragged along. (birth support partner 1).

It was clear from the findings of our study that the women wanted their partners fully involved in the experience of birth, not only because of the support they could provide during labour, but also because of the transitional nature of their relationship – from being a couple to becoming a family. These findings are echoed in the literature [31]. Although there was an initial reluctance from the men in the current study to attend and engage in the classes, this did not last. The aspects of antenatal education that are highlighted in the literature that fail to engage men – a traditional delivery format [32], instruction in what to do, but not being included in a participatory way that enables them to fully provide this support during birth [33], a lack of recognition that they too, have needs during the birthing process; and content not being directed at them to facilitate their involvement [34] were circumvented by the pedagogical approach and content of MBCE, namely changing configurations of dyads and small groups in which men had the opportunity to become knowledge constructers. Similar findings are also noted by Beardshaw [35]. Additionally, all input was listened to, valued and welcomed and voice given to men’s concerns and fears, also described by Friedewald, Fletcher and Fairbairn [36]. As such, MBCE provided a space for men to engage fully in the pending birth of their child.

During labour and birth, it was apparent that the awakened potential of mothers and birth support partners was enacted through being informed, active and questioning in the birth process, and working as a team and staying calm.

### Being an informed, active, questioning participant in the birth process

The knowledge of the birthing process and options for birth, and the confidence mothers and birth support partners gained through the pedagogical approach taken in MBCE enabled them to be active rather than passive participants in the birthing process. The overall effect of this is captured in the following quote from one of the birth support partners:

I think the empowerment for us was we had this knowledge and were confident enough to say to the doctor “let’s wait”… you know, “Can we do this?”. Otherwise if we hadn’t of (sic) gone to this course, I think we would have gone there [hospital] and just like “whatever you say doctor”, you know…“whatever you recommend we’ll do”. This just let us sort of make our, make it feel like it was more our decision than being told what to do. (birth support partner 4).

The overall effect is also captured by a mother:

I was being induced by that Thursday morning and if I hadn’t done this course, like I was booked in and I was in there thinking “Why? Why am I being induced and I didn’t need to be?” And I eventually, actually with the help of my birth partner rang and said “nah, we’re not having the induction” and went into labour spontaneously that night……it [MBCE] probably changed the outcomes of my labour which I was really happy with. (mother 2)

As a result of attending MBCE mothers and birth support partners had the confidence to discuss issues with health professionals and were able to ask and respond in an informed way. For the majority of participants, this resulted in a positive response from health professionals including for example, answering questions and providing information in a truthful and respectful manner:

If she (mother) asked a question they [health professionals during birth] answered it truthfully. So they didn’t leave, even the negative things – like with the epidural “well this can happen, X percentage in every, you know”. So at least there was an awareness. (birth support partner 6).

Additionally, health professionals – particularly midwives – were open to accommodating the needs and wishes of the mothers and birth support partners:

We [mother and birth support partner] had midwives and our midwives were really receptive to what we’d learnt and brought to the table and they were really supportive as well with what we wanted to try. (birth support partner 2).

Thus many women and their birth support partners in this study were treated with respect by caregivers during birth. There is global evidence in the literature that women’s satisfaction with experiences of childbirth is strongly correlated with a woman’s sense of control during labour [1,5,37,38] with this evidence confirmed through metasynthesis [39]. ‘Control’, however, is a complex construct [40] but it appears that the quality of the relationship between the woman and her care-giver and the woman’s involvement in decision making are pivotal [3,39]. Having a relationship where the woman is treated with respect, and involved in decision-making has been shown to override the influences of age, socioeconomic status, ethnicity, preparation for childbirth, the birthing environment including continuity of care, and medical interventions on childbirth satisfaction. Feeling in control of what staff members are doing has also been shown to be associated with lower EPDS scores [4].

It is important to note that the dimension of control that has shown to be most important for satisfaction with childbirth is the one that staff members are most able to facilitate: the way that women perceive they are being treated. That is, women want to be treated with respect and as an individual – as a subject and not an object [8] – and have health professionals understand the importance, for them, of having choice during birth [41] despite not being able to challenge dominant medical discourse [42] and this is reflected in the findings of this research.

A small number of participants in this study, however, described how being informed and confident strained their relationship with attending health professionals:

I was still very flexible and I was open to options, but because my birth plan said only Caesarean in case of emergency my labour team was too scared to offer me a Caesarean almost, because they knew I didn’t want it and because I had, I was never going to be able to deliver naturally. I actually think I scared them off and I can’t help but wonder if I hadn’t been so focussed on that, whether or not I might not have had to have my baby into special care nursery, and all the scheme of events that ended up happening might not have happened. (mother 5).

This finding suggests that, despite the plethora of research highlighting the importance of women being involved in decision making, health professionals may be accustomed to passive and accepting ‘patients’ and find it more challenging when a woman and their birth support partner are more actively engaged in the decisions around the labour and birth process.

Not only those who experienced an uncomplicated labour and a normal vaginal birth felt they were active and well informed in the birthing process. For those mothers whose birth did not proceed as they had hoped - for a number of reasons - also reflected positively on the birthing experience:

I also thought that it [MBCE] made me think about little things that I might not have considered which made my labour experience better….And despite the fact that I really had what most people would say was a horrible labour experience and a very painful labour experience, I still came out of it going “you know what. I’m really glad I had that experience and I feel empowered by that experience”. (mother 5).

Thus, our study provides further evidence for the growing literature on the importance of choice and being in control for satisfaction in the birthing process, irrespective of whether what mothers hoped for in childbirth was realised or not.

Being mindful adds a further dimension to our understanding of the importance of control for satisfaction with childbirth. For the participants, being mindful related to being aware of choices that were made as they progressed through labour.

You can have this idea of what you want, but you need to remain flexible and as long as you are aware of the choices that you’re making as you progress, which is about being mindful and being mindful of this… choice that I’m faced with right now and I can go either way. Even if you’d have to go the way that it wasn’t in your plan, you can come out of it on the other side thinking that I had this really positive experience and I’m really happy with it. (mother 2).

Participants contrasted what they learned at MBCE and how it was delivered, with more traditional hospital-based antenatal classes. They considered that attendance at MBCE provided information that is not often provided during antenatal classes run through hospitals on a range of options they could avail themselves of during the birthing process. Participants were also exposed to different scenarios which may occur during labour and birth and rehearsed how they might respond through the use of role-play and rehearsal.

I attended the [antenatal] classes at [name of hospital]… I found them very superficial … very small snippets of information and not a lot of opportunity to discuss and share different points of view. So I found that this workshop [MBCE] gave me a lot more empowerment and a lot more information about alternate courses of action and different scenarios, so I’d be prepared [during labour]. (mother 3).

The use of experiential learning and the provision of a wide range of information engaged the women’s birth support partners. This pedagogical approach was in contrast to and counteracted some of the weaknesses of more traditional antenatal classes where a lack of discussion or group work was a consequence of their didactic style of delivery [33]. Importantly, MBCE empowered participants by preparing them for how they might respond to a number of issues that they could, potentially, encounter during the birthing process.

There is evidence to support the provision of non-traditional childbirth education programs such as MBCE with the literature suggesting women and their birth support partners are seeking more than the traditional approach to antenatal education and information. For example, participants report wanting a range of teaching approaches, to cater for a range of learning styles [33,43,44]. Additionally participants in MBCE wanted the opportunity to hear details and ask questions, to learn through discussion and be there to support and share with other participants. In a systematic review of peer-reviewed studies addressing women’s views on antenatal education published between 1996 and 2006 it was found that women prefer a small group learning environment which facilitates discussion between participants as well as the educators [45]. Women also preferred receiving information that they could relate to their individual circumstance. These aspects of successful programs are an integral part of the approach taken in MBCE and have also been shown to have a beneficial effect on maternal parenting self-efficacy [42].

### Ability to stay calm and work as a team

The sense of empowerment that resulted from the knowledge and confidence gained during MBCE enabled mothers and their birth support partner to stay calm during labour and birth. As two birth support partners reflected:

I know she [mother] did [felt empowered] which made me just feel calm then. (birth support partner 1).

Another partner echoed this sentiment: 

*In a sense having that calmness, [was due to] just that fact that you had that bank of knowledge*.…*Whereas if you didn’t have the bank of knowledge…then it would have, maybe been worse (birth support partner 6).*

Being mindful and putting into practice the mindfulness techniques taught during MBCE was also seen by participants as supporting them to remain calm during the birthing process:

I used it [mindfulness] during the birth a bit…It just got us to stay calm, especially when things started going a little bit wrong, just reminded us to calm down again and put your head down, calm down again and keep going. (birth support partners 5).

The metaphor of the eye of the storm was used by a number of participants as a way of describing their sense of calm in what they perceived as an ‘out of control’ external environment. The notion of individual control within an uncontrollable environment was broadly discussed by both mothers and birth support partners. For example:

It [mindfulness] provided me with….a sense of calm and a sense of being in control, even though everything around me was out of control. (mother 7).

As the previous quote shows, remaining ‘calm’ is also constructed as being ‘in control’ – an important influence on birth satisfaction. The women reported using the techniques for relief from pain and during contractions – two areas that Green and Baston examined in their study of 1,146 women at one month antenatally and six weeks postnatally [4]. Green and Baston found that being in control of pain during labour was important for satisfaction with birth for primiparas women if they did not feel they were in control of what staff were doing to them during labour, a finding echoed by Christiaens and Bracke [46]. Thus, it could be argued that through the mindfulness techniques taught in MBCE, participants were provided with an extra tool to influence control and therefore, impact positively on their satisfaction with childbirth.

MBCE was undertaken by participants as a couple. This approach ensured that both the mother and the birth support partner had the same knowledge of the birthing process, the same information regarding options and alternatives, had role-played different scenarios that may present themselves during the birthing process and learned the same techniques to remain in the moment. In essence, this approach to the delivery of MBCE supported and equipped the couple to work as a ‘team’ during the birthing process:

definitely, the course really prepared me for all different kinds of situations that presented in [name of mother’s] birth….And I could provide support [to her]. just kept us informed and knowing you know what questions to ask and when. (birth support partner 4).

There is evidence in the literature that where the birthing woman and her birth support partner present as a unified ‘team’, healthcare professionals are more likely to view them as individuals, where the birth support partner has a unique role, and as an interdependent couple [47]. Supporting the woman’s partner as a parent-to-be has also been shown to strengthen childbirth as a mutually shared experience for the couple [48] and potentially result in higher levels of satisfaction with the childbirth experience.

### Challenge of applying mindfulness beyond birth

When it came to early parenting, however, participants reported being less prepared than they were for birth for the challenges they were presented with. As one mother reflected:

Even though my birth experience was horrific I felt empowered through the whole thing. As soon as he was born I lost that kind of empowerment. I didn’t know what was right, what was wrong, what my role was, when to question, when not to question. So I actually found that that almost detracted a little bit from the birth experience and what I learnt through the course. (mother 3).

Mindfulness training was seen as valuable in restoring calmness in the face of an unsettled baby and, as such, an important tool that new parents could use to regain a sense of calm and control:

When the child’s screaming for hours on end, you deal with it a lot better [using mindfulness] than you might have otherwise….Sometimes I can tell what … path I might have gone down otherwise if I hadn’t done it ‘cause there are times where you can be very stressed by a screaming baby. (mother 1).

It was also seen as valuable for being in the moment with a newborn baby. As one mother noted:

I found it [mindfulness] really helpful after I gave birth. For the first six weeks I think, just when he [baby] doesn’t know me and I don’t know him and sitting on the couch, like for hours, in the middle of the night, just then being able to be in that moment and know where I was. That’s when I felt that I used the tools the most. (mother 5).

A number of birth support partners also continued to use mindfulness in the postnatal phase, suggesting MBCE has value beyond birth. As one birth support partner stated:

I think the mindfulness training was…one of the most valuable things. For me that’s one of the things that I feel like you keep after, you sort of keep on using….I tend to use it in other situations as well. (birth support partner 1).

The saliency of mindfulness techniques in the postnatal period is also noted by Duncan and Bardacke [22] who make particular mention of its value in expanding a user’s range of adaptive strategies for coping with stress. For another birth support partner, however, the incorporation of the techniques into everyday life had not been as marked:

I’m normally fairly relaxed …every now and then when I’m eating I’ll try and eat slower. That’s about it. (birth support partner 3).

This suggests that the ongoing use of mindfulness techniques among participants postnatally was not uniform.

### Being in a community of like-minded parents

It was through the interaction that occurred in the MBCE sessions that participants got to know each other and developed bonds with other soon to be parents. From this, a sense of ‘community’ was borne.

I loved hearing other people talking and challenging concepts and I think I got the most out of that, just the group interaction and participation. Yeah, it was really good. (mother 3).

Participants in MBCE valued the opportunity to share experiences and valued the knowledge that came from hearing the views and opinions of others. The interaction was considered particularly useful where individuals or couples were struggling with particular issues, as hearing that others were, or had experienced similar challenges was validating and learning how they overcame them was instructive:

It was interesting to hear either “this is what I’m struggling with”, then you’d kind of , “ok”, then I could think about that, or hear someone say “who’s struggling with this?” and you think “that’s the same - we have” and you kind of feel a bit oh, “ok”. (birth support partner 5).

The opportunity that the MBCE program provided for the sharing of information and experiences for the participants is reflected in the literature. Sharing experiences of pregnancy is argued to promote relationships among women [49,50]. Not feeling alone in the experience, and finding support for needs that women may not have been aware of until the issues were discussed within the group and individual women could relate to them are also noted in the literature [50]. Research findings also suggest that support obtained from others is a major benefit of attending antenatal classes [33].

Hearing that others were experiencing similar challenges was also considered by participants in MBCE to reduce any sense of isolation:

Well you don’t feel isolated. If someone else is burdened, I might completely know what they’re feeling, or whatever. (birth support partner 6).

The pending birth of their baby was appropriately a ‘top-of-mind’ issue for the participants in MBCE and discussions about options for, and opinions about, the place of delivery was raised as valuable by those who had not yet made a decision:

.When we were deciding where to have the baby and then hearing where other people were having babies I think we got better ideas about maybe going elsewhere or other places that, that hearing people’s recommendations and stuff. (birth support partner 2).

Having similar experiences of pregnancy and preparing for birth was the common ground on which participants were able to initially relate to each other and bond, but the connections became social as well. As one participant noted, the bonds were such that 

week after week, you know, it became a bit of a club I suppose. (birth support partner 6).

The sense of community that permeated both the MBCE sessions continued well beyond childbirth. For example, a Facebook site was established which enabled the virtual flow of information and support between mothers and, for one new mother, filled the void after birth until she was able to join a new mothers’ group:

For me my mother’s group hasn’t started and it doesn’t start ‘til next February (2 months hence) so I felt, I have felt very isolated in that. There hasn’t been anyone with a baby the same age as what I’m going through, the same things at the same time so I can say “hey, my baby’s got a rash. What are you guys using? I’m using this … on my baby… is that helping”. And we’ve actually some of us have done that on the Facebook website. Yeah, we’ve been sharing… tips so that has been a good thing. So definitely have now…having like a group website set up I think has been really helpful, ‘cause we’ve shared different tips and things and that’s been helpful for me again. (mother 5).

Research attests to the expansion of social networks that results from attendance at antenatal classes. In a national cohort study of 1,197 participants in Sweden, researchers found that, although participation in antenatal classes had no impact on first-time mothers’ experiences of childbirth, they were instrumental in expanding the women’s social network [16]. The existence of a social network that extends beyond birth, as was facilitated by attendance at MBCE provides an opportunity for issues related to parenting and life transitions to be discussed within an environment in which participants are already familiar and comfortable.

## Conclusions

Through the innovative combination of MBSR and a pedagogical approach that encompassed role-playing and discussion, MBCE participants felt empowered during birth and able to maintain a sense of control even if the birth did not proceed as planned. A sense of being in control was apparent both for birthing mothers and their birth support partners. Thus, MBCE has the potential to address common concerns in the literature regarding the relationship between a sense of control and satisfaction with the birthing experience. It also has potential to engage the birthing mother’s birth support partner in a meaningful way in antenatal education and birth. The sense of community that developed across the program was strong and was sustained into the postnatal period. This enabled the new parents to continue to support each other during early parenting – another area highlighted in the literature that traditional antenatal education inadequately addresses.

The experiences of participants in this study suggest that health care professionals generally responded positively to them, when proposed procedures were questioned or more information was sought. This, however, was not always the case indicating that further professional development and education should be undertaken with healthcare professionals. Because health care professionals operate in line with organisational policies and culture, work undertaken at the institutional level is also necessary to ensure that birthing women and their birth support partners are listened to and have their input valued, that is, an experience that becomes the norm across healthcare settings.

The findings reported have limitations in that they are from a pilot study with participants homogenous in terms of their socio-demographic characteristics and geographic location. Therefore, further research is required on a larger scale and across a more diverse cohort of expectant mothers, including multiparous women, and their birth support partners to provide stronger empirical evidence of the efficacy and effectiveness of MBCE in terms of facilitating satisfaction and a positive birth experience for the childbearing women and their birth support partners.

## Abbreviations

MBCE: Mindfulness-Based Childbirth Education; MBSR: Mindfulness-Based Stress Reduction.

## Competing interests

The authors declare they have no competing interests.

## Authors’ contributions

CF – facilitated focus group, analysed data, drafted manuscript, YH – facilitated focus group, analysed data, provided critical feedback on manuscript, SB – analysed data, provided critical feedback on manuscript, JB – analysed data, provided critical feedback on manuscript. All authors read and approved the final manuscript.

## Authors’ information

CF has a background in sociology is a teaching and research academic in the School of Population Health at the University of Western Australia. Her main research is in the area of psychosocial women’s health.

YH is the Professor of Midwifery, a joint appointment between Curtin University and King Edward Memorial Hospital. Her research interests include antenatal education, perinatal mental health and breastfeeding.

SB is a midwifery clinician and academic. The current focus of her scholarly work is on organisational learning in relation to innovation and the adoption of evidence in healthcare settings.

JB is a childbirth educator and specialist pregnancy yoga teacher and trainer. Her research focus on mindfulness, modern yoga studies and feminist philosophy.

## Pre-publication history

The pre-publication history for this paper can be accessed here:

http://www.biomedcentral.com/1471-2393/12/126/prepub
